# Polymer-based controlled-release fed-batch microtiter plate – diminishing the gap between early process development and production conditions

**DOI:** 10.1186/s13036-019-0147-6

**Published:** 2019-02-22

**Authors:** T. Keil, B. Dittrich, C. Lattermann, T. Habicher, J. Büchs

**Affiliations:** 1 AVT - Biochemical Engineering, RWTH Aachen University, Forckenbeckstraße 51, 52074 Aachen, Germany; 2 DWI – Leibniz Institute for Interactive Materials, RWTH Aachen University, Forckenbeckstraße 50, 52074 Aachen, Germany; 3 Kuhner Shaker GmbH, Kaiserstraße 100, 52134 Herzogenrath, Germany

**Keywords:** Fed-batch, Microtiter plate, *Escherichia coli*, *Hansenula polymorpha*, Screening, High-throughput, Bioprocess development

## Abstract

**Background:**

Fed-batch conditions are advantageous for industrial cultivations as they avoid unfavorable phenomena appearing in batch cultivations. Those are for example the formation of overflow metabolites, catabolite repression, oxygen limitation or inhibition due to elevated osmotic concentrations. For both, the early bioprocess development and the optimization of existing bioprocesses, small-scale reaction vessels are applied to ensure high throughput, low costs and prompt results. However, most conventional small-scale procedures work in batch operation mode, which stands in contrast to fed-batch conditions in large-scale bioprocesses. Extensive expenditure for installations and operation accompany almost all cultivation systems in the market allowing fed-batch conditions in small-scale. An alternative, more cost efficient enzymatic glucose release system is strongly influenced by environmental conditions. To overcome these issues, this study investigates a polymer-based fed-batch system for controlled substrate release in microtiter plates.

**Results:**

Immobilizing a solid silicone matrix with embedded glucose crystals at the bottom of each well of a microtiter plate is a suitable technique for implementing fed-batch conditions in microtiter plates. The results showed that the glucose release rate depends on the osmotic concentration, the pH and the temperature of the medium. Moreover, the applied nitrogen source proved to influence the glucose release rate. A new developed mathematical tool predicts the glucose release for various media conditions. The two model organisms *E. coli* and *H. polymorpha* were cultivated in the fed-batch microtiter plate to investigate the general applicability for microbial systems. Online monitoring of the oxygen transfer rate and offline analysis of substrate, product, biomass and pH confirmed that fed-batch conditions are comparable to large-scale cultivations. Furthermore, due to fed-batch conditions in microtiter plates, product formation could be enhanced by the factor 245 compared to batch cultivations.

**Conclusions:**

The polymer-based fed-batch microtiter plate represents a sophisticated and cost efficient system to mimic typical industrial fed-batch conditions in small-scale. Thus, a more reliable strain screening and early process development can be performed. A systematical scale-down with low expenditure of work, time and money is possible.

**Electronic supplementary material:**

The online version of this article (10.1186/s13036-019-0147-6) contains supplementary material, which is available to authorized users.

## Background

The range of products based on microbiological production processes increase significantly with advances in modern biomolecular engineering. Bulk chemicals, for example bioethanol [1] or 2,3-butandiol [2], are fabricated based on microbial cultivation processes as well as highly complex, pharmaceutical active substances, for example insulin [3]. When working with microbial production systems in industrial scale, fed-batch operation mode appears to be beneficial in comparison to a standard batch cultivation. In this work, fed-batch operation is defined as the continuous feeding of a substrate to a culture in a way that the cultivated microorganisms immediately take up the supplied substrate. Fed-batch cultivations avoid the disadvantageous effect of overflow metabolism, catabolite repression or oxygen limitation appearing in batch cultivations [4–8]. By this means, high biomass yields and an effective production of various biomolecules is achieved.

The development of such bioprocesses is commonly based on a trial and error strategy due to the large number of variables relevant in biotechnological processes [9]. Hence, almost all process development occurs in small-scale cultivation vessels with a volume of few microliters to allow high-throughput procedures [10]. As a standard cultivation vessel, microtiter plates are applied to conduct high numbers of experiments in a very compact and standardized platform [11]. Much effort has been put into developing technologies for online monitoring of meaningful cultivation data by measuring backscattered light, fluorescence [12], pH [13], dissolved oxygen tension [14, 15] or oxygen transfer rates [16] in microtiter plates. Moreover, advanced techniques allow automated bioprocess development since liquid handling systems can perform a multitude of manipulations like adding inductors or withdraw samples [17–19].

To develop a successful bioprocess consistently and to gain scalable results, it is essential to imitate large-scale production conditions already in small-scale cultivations [9, 20]. For instance, strain candidates performing promisingly in conventional batch microtiter plates will disappoint during scale-up in fed-batch conditions. Even worse, optimal candidates for fed-batch conditions will not be identified in the screening [21]. Furthermore, information acquired about cultivation conditions during batch screening experiments is not directly transferable for further process development in fed-batch. These arguments reveal the necessity of applying fed-batch operation mode already in small-scale cultivation vessels like microtiter plates.

Implementing fed-batch conditions in small-scale cultivation vessels has been attempted in various ways in the last years. Puskeiler et al. introduced a miniature stirred tank bioreactor system with a cultivation volume of 10 mL per reactor [22]. The feeding is realized by pipetting a substrate solution with a liquid handling system into the mini-reactors. This system can be extended by the integration of magnetic micro pumps, enabling a continuous supply of feed solution [23]. Other stirred micro bioreactor systems like Micro-24 (Pall Corporation, New York, USA), Micro-matrix (Applikon Biotechnology, Delft, Netherlands) or Ambr (Sartorius AG, Göttingen, Germany) provide similar functionalities [24–26]. Nevertheless, these systems use specific miniaturized cultivation vessels that do not allow an easy integration into existing microtiter plate handling systems. An alternative approach bypassing this drawback is the microfluidic microtiter plate system BioLector Pro (m2p-labs GmbH, Baesweiler, Germany) [27–29]. Here, feeding is realized by micro pumps transferring substrate solution from a reservoir well into the cultivation well of a 48-round-well microtiter plate. However, for all mentioned systems, considerable investment and operational costs accrue and the integration into existing microtiter plate handling systems is not directly possible [26].

A sophisticated way to realize fed-batch conditions independently from scale and cultivation vessel is proposed by Panula-Perälä et al. [30]. Glucose is enzymatically released by the degradation of starch applying glucoamylases from *Aspergillus niger*. Thus, a continuous release of glucose is realized and fed-batch conditions are established [31–33]. The glucose release rate can be adjusted by the amount of added enzyme. However, biological systems producing proteases or intrinsic glucoamylases lead to an uncontrolled glucose release. Additionally, cultivation conditions like pH or temperature strongly influence the enzyme activity [34]. The limitation to glucose as sole feeding substrate further restricts the applicability of this technique.

**Fig. 1:**
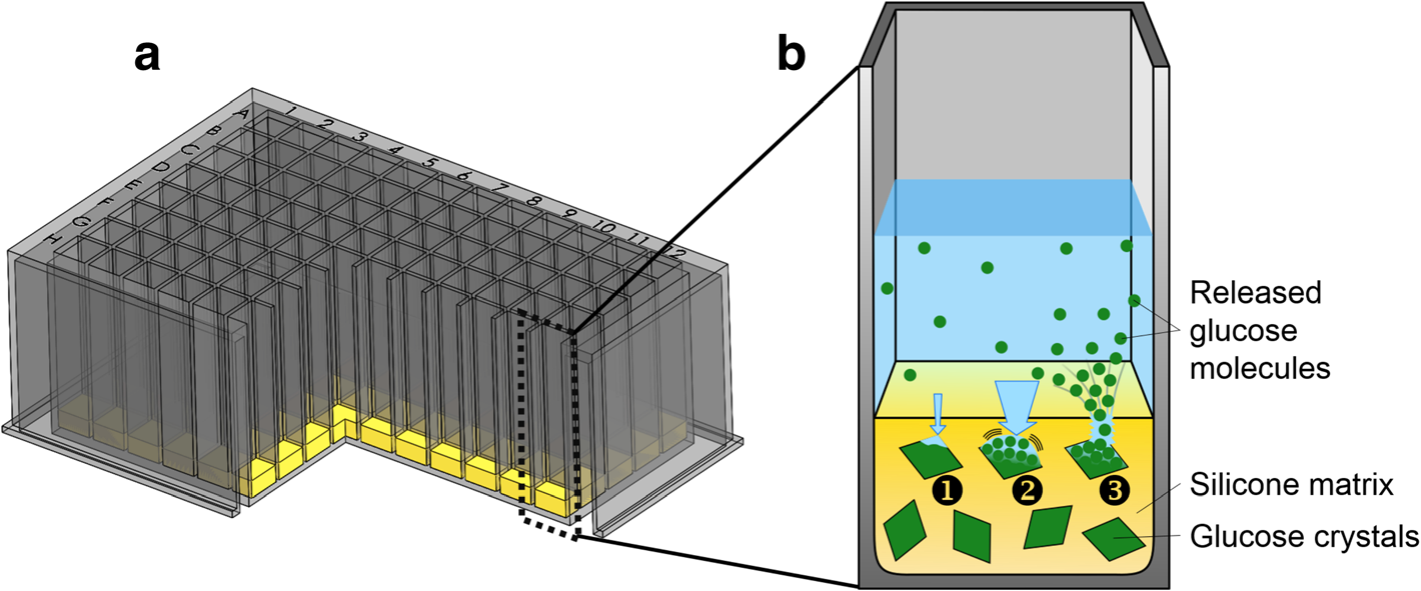
Principle of fed-batch microtiter plate (FeedPlate®). **a** Cross section view of a 96-square-well fed-batch microtiter plate with the release system at the bottom of each well. **b** Enlarged cross section view of one well (not in scale). Embedded glucose crystals inside of the silicone matrix serve as nutrient reservoir. The mechanism of the glucose release is illustrated: 1) Water diffuses into the silicone matrix and creates a small pore with highly concentrated glucose solution. 2) Due to osmotic pressure, more water is forced into the cavity. 3) At some point, cracks and channels are established in the silicone matrix, which is followed by the release of the glucose solution into the culture medium. For release and cultivation experiments, the microtiter plate was placed in a temperature and humidity controlled incubation hood and shaken at 970 rpm with 3 mm shaking diameter

An alternative system described by Jeude et al. releases nutrients from a silicon elastomer matrix [35]. Several successful cultivations with suspended silicon disks in shake flask have been reported [35–38]. Furthermore, cultivations were conducted with discs fixated at the bottom of a 96-round-well microtiter plate [39]. Recently, the idea has been extended to a ready to use microtiter plate with an immobilized release system at the bottom of each well visualized in Fig. 1A (FeedPlate®, Kuhner Shaker GmbH, Herzogenrath, Germany). The release mechanism from the silicone matrix is a sequential, osmotic process as depicted in Fig. 1B. First, water diffuses into the matrix and dissolves crystalline glucose. This step creates little liquid volumes of highly concentrated glucose solution. Second, due to osmotic pressure, more water is forced into the cavity. Third, the swelling exceeds the elastic elongation of the matrix and micro-channels are established. Through this network, the substrate is released into the medium. It is possible to release almost all water-soluble solids with this system. No further technical investment is necessary, since the microtiter plate design remains unchanged.

This study investigates the glucose release of the commercially available polymer-based fed-batch microtiter plate into various, commonly applied media. The influence of cultivation conditions on the glucose release is systematically evaluated. Therefore, various parameters like osmotic concentration, pH, temperature and the type of applied salt-compounds have been considered. A predictive tool for estimating the total amount of released glucose into a defined mineral medium in dependency of the mentioned parameters is presented. Microbial cultivations with the prokaryotic organism *E. coli* producing a flavin mononucleotide binding fluorescence protein (FbFP) and with the eukaryotic organism *H. polymorpha* producing green fluorescent protein (GFP) were conducted. Important process parameters, including the oxygen transfer rate, were investigated while applying the fed-batch microtiter plate.

## Material and methods

### Microtiter plates

For fed-batch cultivations, 96-square-well (FeedPlate®, Part number: SMFP08002, Kuhner Shaker GmbH, Herzogenrath, Germany) and 48-round-well (prototype) microtiter plates were used. At the bottom of each well, a release system is located, which consists of a cross-linked siloxane elastomer with embedded glucose crystals. The cross-linking is catalyzed by a Karstedt’s catalyst. The embedded glucose serves as a reservoir and is released by an osmotic driven mechanism. For the prototype, the permeation capability of the siloxane matrix for water is enlarged by the addition of hydrophilic siloxane copolymers. For batch cultivation, 96-square-well (Part number: 850301, HJ-BIOANALYTIK GmbH, Erkelenz, Germany) and 48-round-well microtiter plates (MTP-R48-OFF, m2p-labs GmbH, Beasweiler, Germany) were used.

For all microtiter plate cultivations and glucose release experiments in 96-square-well plates, “AeraSeal Film” (A9224, Sigma-Aldrich Chemie GmbH, Germany) sealings were used as permeable sterile barrier [40]. For release experiments at 45 °C, an airtight, self-made silicone sealing was fixed on top of the plate. The whole setup (microtiter plate and sealing) was placed inside the cultivation hood. “Polyolefin sealing foil” (900371-T, HJ-Bioanalytik GmbH, Erkelenz, Germany) was used for cultivations in 48-round-well plates.

### Media

All components were purchased from either Carl Roth GmbH & Co. KG (Karlsruhe, Germany), Sigma Aldrich Chemie GmbH (Steinheim, Germany) or Merck KGaA (Darmstadt, Germany) if not otherwise stated.

In this work, all *E. coli* cultivations (preculture and main culture) were conducted in Wilms-MOPS medium. The base solution contains 6.98 g/L (NH_4_)_2_SO_4_, 3 g/L K_2_HPO_4_, 2 g/L Na_2_SO_4_, 41.85 g/L (N-morpholino)-propane sulfonic acid (MOPS, 0.2 M) and 0.5 g/L MgSO_4_ × 7 H_2_O. pH was adjusted to a value of 7.5 with 10 M NaOH solution. Before each microbial cultivation experiment, 1 mL/L of a 10 g/L thiamine chloride hydrochloride stock solution and 1 mL/L trace element solution were added (both sterile filtered). The trace element solution consists of 0.54 g/L ZnSO_4_ × 7 H_2_O, 0.48 g/L CuSO_4_ × 5 H_2_O, 0.3 g/L MnSO_4_ × H_2_O, 0.54 g/L CoCl_2_ × 6 H_2_O, 41.76 g/L FeCl_3_ × 6 H_2_O, 1.98 g/L CaCl_2_ × 2 H_2_O, 33.4 g/L Na_2_EDTA (Titriplex III, Merck, Darmstadt, Germany). For cultivation experiments, 20 g/L glucose (batch) or no glucose (fed-batch) was initially added to the medium.

Glucose release experiments using the Wilms-MOPS medium were conducted solely with the base solution (without glucose). Except for the respective investigated parameter, all other parameters were kept constant. Different osmotic concentrations were realized by applying several MOPS concentrations (between 0 and 200 mM). Additionally, different pH values (between 4.5 and 8), temperatures (25, 37, 45 °C) and nitrogen sources (Urea, KNO_3_, NH_4_NO_3_, NH_4_Cl, (NH_4_)_2_SO_4_) were analysed. For the latter, the molarity of nitrogen (105 mmol_N_/L) was constant for all nitrogen sources. Additionally, various ammonium sulphate concentrations (between 0 and 105 mM) were investigated. To analyse the glucose release into media with pH 5 and 4.5, MOPS buffer was replaced equimolar by 2-(N-morpholino)-ethane sulphonic acid (MES) buffer.

In this work, all *H. polymorpha* cultivations (preculture and main culture) were conducted with Syn6-MES medium. The base solution contains 1.0 g/L KH_2_PO_4_, 7.66 g/L (NH_4_)_2_SO_4_, 3.3 g/L KCl, 3.0 g/L MgSO_4_ × 7 H_2_O, 0.3 g/L NaCl, 27.3 g/L 2-(N-morpholino)-ethane sulphonic acid (MES). pH was adjusted to a value of 6.0 with 10 M NaOH solution. Before each microbial cultivation experiment, 10 mL/L of 100 g/L CaCl_2_ × 2H_2_O, 10 mL/L microelements, 10 mL/L vitamin solution and 10 mL/L trace element solution (all sterile filtered) were added. The microelement solution consists of 6.65 g/L (NH_4_)_2_Fe(SO_4_)_2_ × 6 H_2_O, 0.55 g/L CuSO_4_ × 5 H_2_O, 2.0 g/L ZnSO_4_ × 7 H_2_O, 2.65 g/L MnSO_4_ × H_2_O, 6.65 g/L Na_2_EDTA (Titriplex III, Merck, Darmstadt, Germany). The vitamin solution consists of 0.4 g/L D-Biotin and 13.35 g/L thiamine chloride hydrochloride. The trace element solution consists of 0.065 mg/L NiSO_4_ × 6 H_2_O, 0.065 mg/L CoCl_2_ × 6 H_2_O, 0.065 mg/L H_3_BO_3_, 0.065 mg/L KI, 0.065 mg/L Na_2_MoO_4_ × 2 H_2_O. For cultivation experiments, 10 g/L glucose (batch) or no glucose (fed-batch) was initially added to the medium. The glucose release experiments into this medium were conducted solely with the Syn6-MES base solution; in particular, no glucose was added to the medium.

Phosphate buffered saline (PBS) tablets were used (Part number: A9191, AppliChem GmbH, Darmstadt, Germany) to prepare PBS medium. Lysogeny broth (LB) medium, terrific broth (TB) medium and yeast extract peptone medium (YP) medium are complex media. LB medium consists of 10 g/L tryptone (Part number: 8952.4; Carl Roth GmbH & Co. KG, Karlsruhe, Germany), 5 g/L yeast extract (Part number: 2904.4; Carl Roth GmbH & Co. KG, Karlsruhe, Germany) and 5 g/L NaCl. TB medium consists of 12 g/L tryptone, 24 g/L yeast extract, 12.54 g/L K_2_HPO_4_ and 2.31 g/L KH_2_PO_4_. YP medium consists of 20 g/L tryptone and 10 g/L yeast extract. To avoid unintended growth of contaminants during the course of all release experiments, 0.2 g/L NaN_3_ was added to each medium.

### Determination of glucose release kinetics

Glucose was determined spectrometrically applying an enzymatic assay using glucose-6-phosphat-dehydrogenase and hexokinase based on the approach described by Slein [41]. Each glucose data point represents the mean value of three individually analysed wells. Evaporation was measured gravimetrically and data points were corrected correspondingly. The glucose release rates (*v*) were defined as the slope of the linear regression of the glucose release over time. Data points enclosed in brackets in Fig. 3 were neglected for the regression since a saturation occurs at released glucose amounts higher than 13 mg in ammonia containing media. During the release experiments, the plates were shaken in a cultivation hood (ISF1-X, Kuhner Shaker GmbH, Herzogenrath, Germany) at a frequency of 970 rpm at a shaking diameter of 3 mm. The cultivation hood is capable of maintaining temperatures up to 80 °C. The filling volume per well always was 1000 μL at a humidity of 80%.

### Microorganisms

*Escherichia coli* BL21 (DE3) pRhotHi-2-EcFbFP (GenBank Number: ABN71355) [42] was used as a prokaryotic model organism in this study. The strain produces a flavin mononucleotide binding fluorescence protein (FbFP) under the control of the lac operator [18, 43]. *Hansenula polymorpha* RB11 pC10-FMD (P_FMD_-GFP) [39] was used as a eukaryotic model organism. The strain produces a green fluorescence protein (GFP) under the control of a FMD promoter.

### Cultivation procedure

All cultivations were conducted in a cultivation hood (ISF1-X, Kuhner Shaker GmbH, Herzogenrath, Germany), providing shaking of the cultivation vessels and constant environmental conditions (temperature and humidity). For preculture, a 250 mL shake flask was filled with 10 mL medium (for *E. coli*: Wilms-MOPS with 20 g/L glucose; for *H. polymorpha*: Syn6-MES with 10 g/L glucose). The culture was inoculated from a cryo-stock and cultivated for 17 h at 30 °C on an orbital shaker at a shaking frequency of 300 rpm and a shaking diameter of 50 mm. The main culture was inoculated with centrifuged and resuspended cells from the preculture to an initial biomass concentration of 0.11 g/L for *E. coli* (Wilms-MOPS) and 0.35 g/L for *H. polymorpha* (Syn6-MES). Subsequently, each well of a 96-square-well microtiter plate was filled with 600 μL of inoculated medium. The general cultivation conditions were: temperature = 37 °C; shaking frequency: *n* = 970 rpm; shaking diameter: d = 3 mm; humidity = 80%. For *E. coli*, the initial pH was 7.5. For *H. polymorpha*, the initial pH was 6.0. For cultivation in the prototype 48-round-well fed-batch microtiter plates, all parameters were identical to those cultivations in 96-square-well plates, but the filling volume: V_L,48_ = 800 μL/well.

### Online measurements of the oxygen transfer rate in microtiter plates

The oxygen transfer rate (OTR) was measured quasi-continuously following the measuring principle of Anderlei et al. [44, 45]. In this study, the OTR was determined for a whole 96-square-well microtiter plate [46, 47]. This means, the measured value is an averaged value over 96 wells (replicates) and therefore, each well has to be filled with the same volume. The respiration quotient (RQ) was calculated following Anderlei et al. [45]. For the newly developed 48-round-well fed-batch microtiter plate the technique described by Flitsch et al. [16] was applied to measure the OTR in each individual well.

### Offline sample analysis

pH was measured in the supernatant with HI2211 Basic pH / Redox / °C Meter (Hanna Instruments, Vöhringen, Germany). Osmotic concentration was determined with an Osmomat 3000 basic (Gonotec GmbH, Berlin, Germany). Prior to measurement, a three-point calibration with DI-water and calibration standards (500 and 850 mOsmol/kg) was conducted. The cell dry weight was derived from optical density measurements. The optical density was measured at a wavelength of 600 nm (OD_600_) with a plate reader (Synergy 4 Microplate Reader, BioTek, Winooski, VT, USA). For calibration, cell dry weight was determined gravimetrically in centrifuge tubes. For *E. coli* 1 g/L biomass corresponds to an OD_600_ of 1.75, for *H. polymorpha* 1 g/L biomass is equivalent to an OD_600_ of 1.41. GFP fluorescence intensity was measured with an excitation wavelength of 485 nm and an emission wavelength of 520 nm in the plate reader. The GFP-yield was calculated by dividing the GFP value with the respective amount of provided glucose at that point of time. Measurements of glucose and acetate from cultivation samples were performed by high-performance liquid chromatography (Prominence HPLC, Shimadzu Deutschland GmbH, Duisburg, Germany), using an ion-exclusion column (ROA-Organic Acid H+; Phenomenex Inc.; Aschaffenburg; Germany) and a refractometer for detection (RID-20A; Shimadzu Deutschland GmbH, Duisburg, Germany). For the measurement, supernatant of the samples was filtered (pore size: 0.2 μm), diluted to an appropriate concentration range, and stored at 4 °C. The measurement was conducted with 5 mM sulphuric acid solution as eluent, at a flow rate of 0.8 mL/min and a temperature of 60 °C.

## Results and discussion

### Glucose release into commonly applied media

**Fig. 2:**
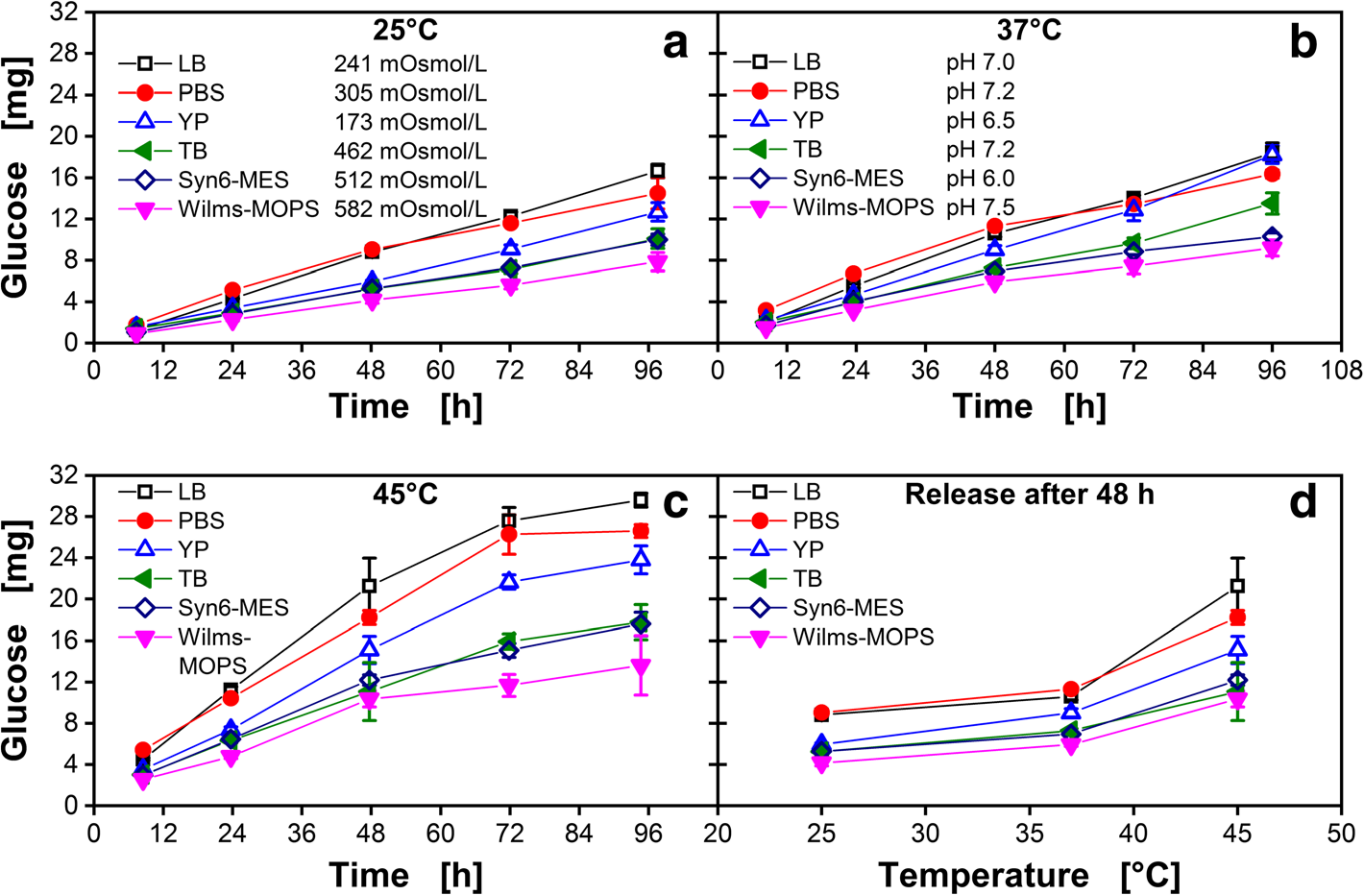
Amount of glucose released per well over time of a 96-square-well polymer-based fed-batch microtiter plate. Glucose release into a selection of commonly applied complex (LB, YP, TB) and synthetic media (PBS, Syn6-MES, Wilms-MOPS) is depicted. All data points are mean values of measurements of three individual wells. Error-bars indicate the respective standard deviation. **a** 25 °C **b** 37 °C **c** 45 °C **d** Temperature dependency of the amount of released glucose in all six investigated media after 48 h. Experimental conditions: Humidity = 80%, shaking frequency = 970 rpm, shaking diameter = 3 mm, V_L,96_ = 1000 μL/well. To avoid unintended growth of contaminants, 0.2 g/L NaN_3_ was added to the medium

The general glucose release into different media at different temperatures was examined in Fig. 2. Glucose release over time for three different complex media (LB, YP, TB) and three different synthetic media (Wilms-MOPS, Syn6-MES, PBS) at 25 °C (Fig. 2A), 37 °C (Fig. 2B) and 45 °C (Fig. 2C) are displayed. Glucose is continuously released from the matrix into each medium since the glucose amount increases over time. The glucose release increases with temperature (Fig. 2D). The glucose release rates elevate from 25 to 37 °C by 23% and from 37 to 45 °C by 63%. While the release follows an almost linear manner for 25 °C and 37 °C (Fig. 2A and B), the release declines at longer release times for 45 °C (t > 72 h; Fig. 2C). For all temperatures, LB and PBS medium show the highest glucose release over time. Glucose release into YP medium is reduced, whereas TB and Syn6-MES medium show relatively low glucose release. However, all above-mentioned media reveal a higher glucose release than Wilms-MOPS medium. Considering the small error bars (standard deviation of 4.5%, each data point corresponds to three individual wells) and constant release of glucose, the fed-batch microtiter plate is working properly and reproducible. However, there are influencing factors manipulating the glucose release that need to be investigated.

### Influencing factors on glucose release

**Fig. 3:**
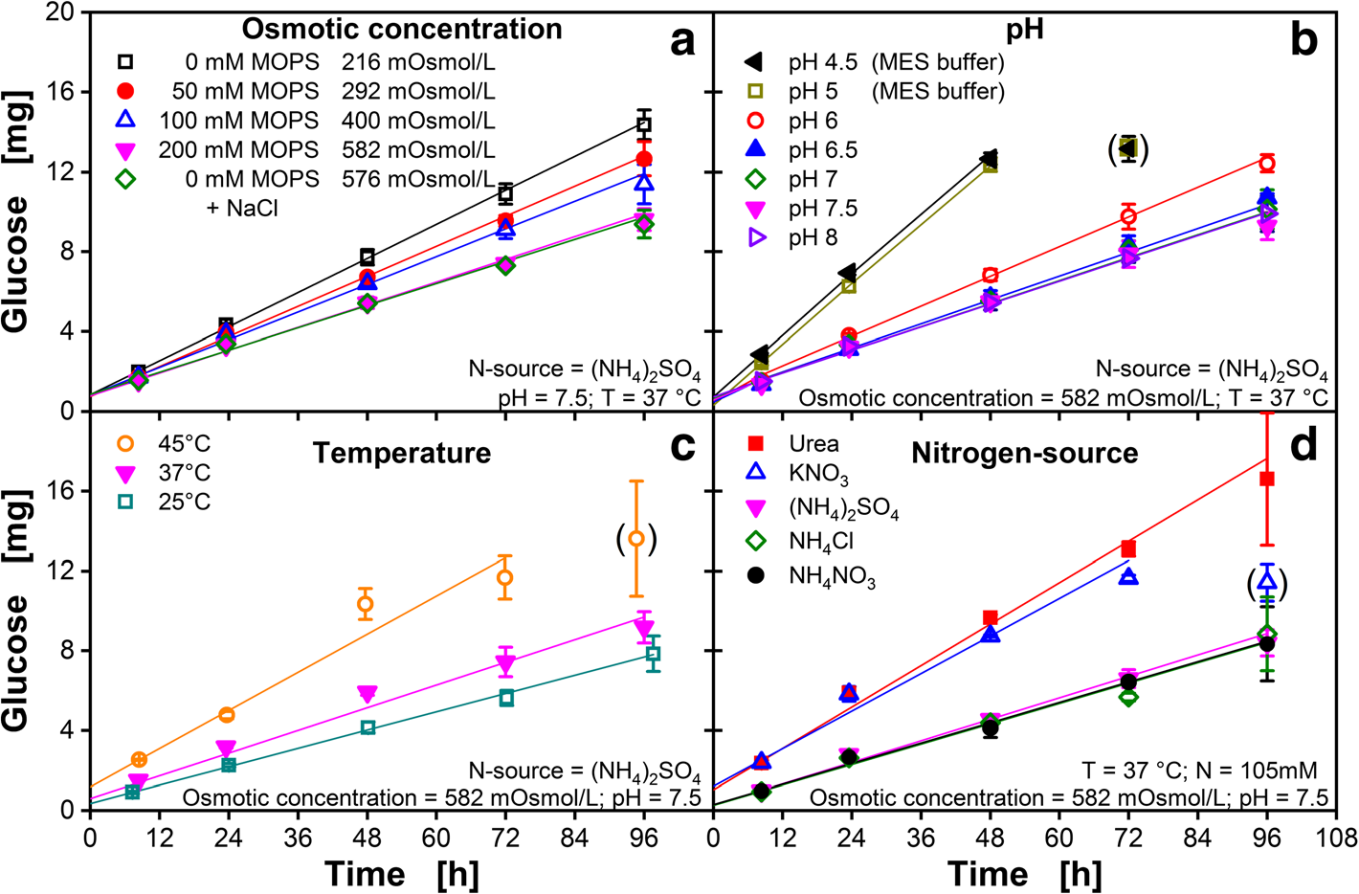
Amount of glucose released per well of a 96-square-well fed-batch microtiter plate into Wilms-MOPS medium. Lines indicate linear fits for each condition. Data points in brackets are not considered for fitting. All data points are mean values of measurements of three individual wells. Error-bars indicate the respective standard deviation. Except for the investigated parameter, all other parameters were kept constant as indicated at the bottom of each subfigure. **a** Influence of varying osmotic concentration on the total amount of glucose released by changing the MOPS buffer concentration (0, 50, 100, 200 mM). Additionally, 200 mM MOPS is replaced by NaCl to rule out any influence of MOPS itself on the glucose release. **b** Influence of varying pH-values on the total amount of glucose released. For the pH range from 6 to 8, 200 mM MOPS buffer was applied, for pH 4.5 and 5, 200 mM MES buffer was applied. **c** Influence of varying temperature (25, 37, 45 °C) on the total amount of glucose released. **d** Influence of varying nitrogen-sources (Urea, KNO_3_, NH_4_NO_3_, NH_4_Cl, (NH_4_)_2_SO_4_) on the total amount of glucose released. The molarity of nitrogen was constant (105 mM_N_) for all nitrogen sources. General experimental conditions: Humidity = 80%, shaking frequency = 970 rpm, shaking diameter = 3 mm, V_L,96_ = 1000 μL/well. No initial glucose was provided in the medium. No biology was applied. To avoid unintended growth of contaminants, 0.2 g/L NaN_3_ was added to the medium

For further evaluation of the fed-batch microtiter plate, modified Wilms-MOPS medium is applied as standard medium. In Fig. 3 the total amount of glucose released over time is analyzed regarding osmotic concentration, pH, temperature and the applied nitrogen source.

Figure 3A shows the dependency of the glucose release on the osmotic concentration of the medium in the range between 216 and 582 mOsmol/L. The highest glucose release was found with 0 mM MOPS buffer (216 mOsmol/L), whereas the lowest release was identified with 200 mM MOPS (582 mOsmol/L). Apparently, the lower the osmotic concentration is in the medium, the higher is the glucose release. This finding is reasonable, since the osmotic pressure is a major driving force for the release mechanism. The osmotic concentration of the medium was adjusted with sodium chloride (green, diamonds) to a level comparable to the 200 mM Wilms-MOPS medium. There is no difference in the glucose release between these solutions. This proves that indeed the osmotic concentration and not the chemical nature of the MOPS buffer is the cause for the lower glucose release

The glucose release into Wilms-MOPS medium at different pH values is depicted in Fig. 3B. For pH values between 6.5 and 8, no substantial influence on the glucose release can be identified. At pH values below 6.5, the glucose release increases significantly, but remains relatively constant for values lower than 5. Release experiments with MOPS and MES buffer, both at pH 6, showed that the type of buffer is not influencing the glucose release (data not shown). The varying glucose release behavior with changing pH in the culture broth might become important if the investigated strain produces a substantial amount of acidic compounds. In the optimal pH range for bacterial strains (6.5–7.5 [48, 49]), the glucose release is relatively constant and shows a variation of less than 10%. In comparison, the enzymatic degradation of a glucose polymer by glucoamylases as an alternative fed-batch system showed an increase of glucose release of 40% when changing the pH from 7.3 to 6.8 [34].

In Fig. 3C, the glucose release at different temperatures is presented. Higher temperatures lead to increased glucose release rates, which is in accordance to Fig. 2. The first step of the release process (depicted in Fig. 1B) is diffusion controlled. Thus, the rate of diffusion depends on the applied temperature [50] and the observed correlation between glucose release and temperature is reasonable. In comparison, the enzymatic system published by Panula-Perälä et al. [30] shows an 76% higher glucose release at 37 °C compared to the glucose release at 25 °C [34]. The fed-batch microtiter plate shows a variation of less than 24% in the same temperature range.

Figure 3D depicts the glucose release into Wilms-MOPS medium with different nitrogen sources. All ammonia containing media show comparable glucose release over time, regardless of the corresponding anion of the salt. In media with alternative nitrogen sources, like potassium nitrate or urea, a significantly higher glucose release was observed. A reaction between glucose and ammonia to products that are not detected or distinguished by enzymatic glucose assays or HPLC could explain this phenomenon. It has already been reported that glucose and aqueous ammonia are capable of forming glucosamines or imidazole compounds [51, 52]. High glucose concentrations inside the matrix and the presence of the Karstedt’s catalyst might promote reactions forming these products [53]. This assumption is supported by an increase in absorbance of the media at wavelengths between 300 and 400 nm in long-term release experiments, which is typical for reaction products of hexoses and amino components [54].

For a deeper understanding, Additional file 1 depicts the glucose release into media containing various ammonia concentrations. For medium without ammonia (0 mM), 7 mg and 11 mg glucose were released per well after 24 and 48 h, respectively. These values are similar to the amounts of released glucose into media containing other nitrogen sources (urea and KNO_3_) after 24 h (~ 7 mg; Fig. 3D) and 48 h (~ 11 mg; Fig. 3D). However, with increasing ammonia concentration, less glucose can be detected (Additional file 1; range 0–20 mM ammonia). As soon as the ammonia concentration exceeds a specific threshold value of roughly 20 mM, the glucose release rate appears to be constant (Additional file 1; range 20–105 mM ammonia).

As a general result, Figs. 2 and 3 and Additional file 1 confirm a linear glucose release for almost all applied conditions. Nevertheless, for released glucose amounts higher than 13 mg (data points in brackets in Fig. 3B and C), a saturation is noticed for standard Wilms-MOPS medium. Linear regressions of all glucose release data over time in Fig. 3A - D reveal that the y-intercept of most of these fits does not match with the origin. This is attributed to a relatively high initial glucose release, which is inherent for this kind of polymer-based material. The mean y-intercept value is m_init_ = 0.901 mg.

### Modeling and prediction of the glucose release

**Fig. 4:**
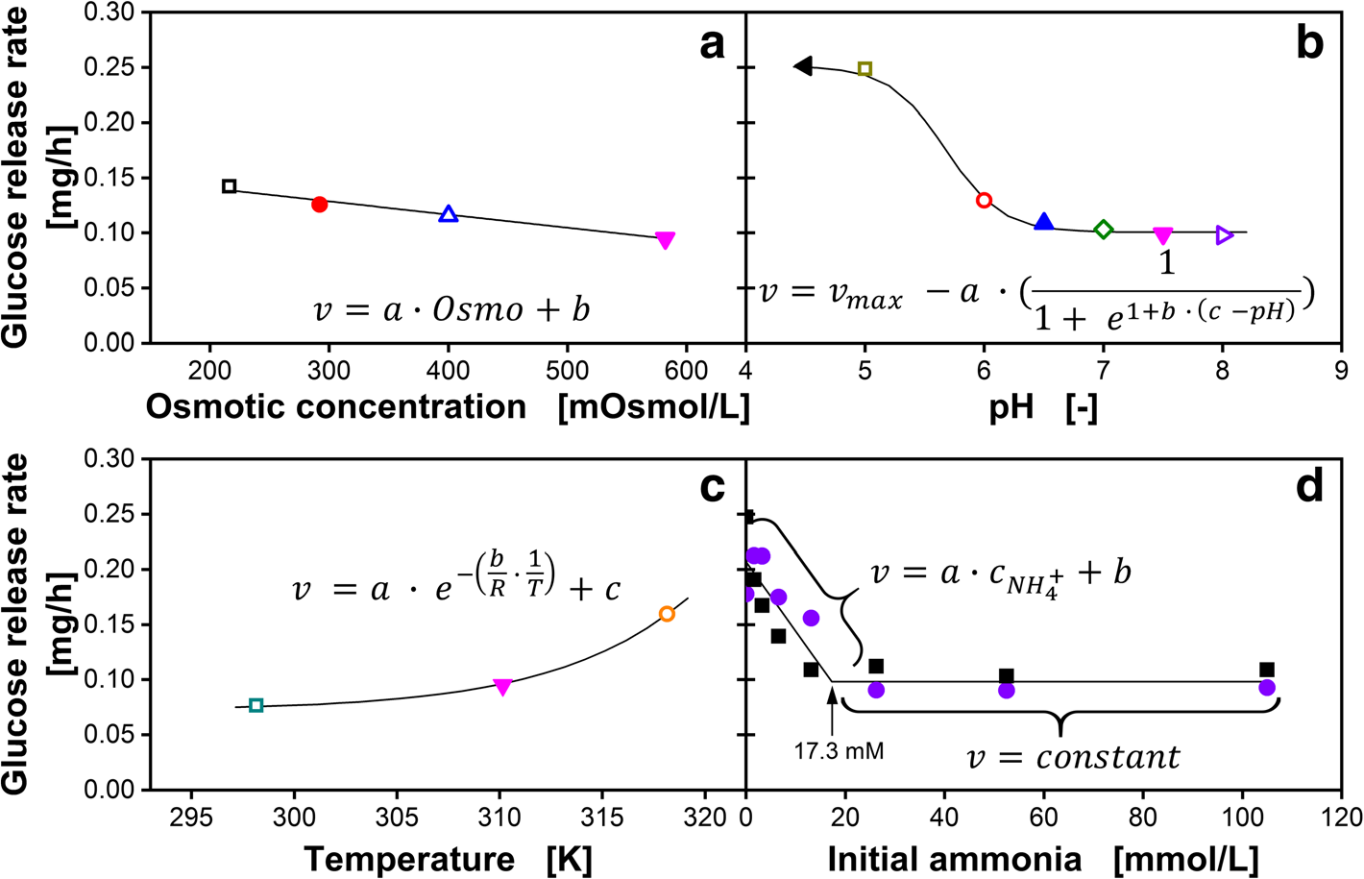
Glucose release rate per well of a 96-square-well fed-batch microtiter plate into Wilms-MOPS medium. For **a**, **b** and **c** each data point corresponds to the slope of each linear fit from Fig. 3a, b and c, respectively. For **d** the data points correspond to the glucose release derived from Additional file 1. Except for the investigated parameter, all other parameters were kept constant. The solid lines represent the respective equation. **a** Glucose release in dependency of osmotic concentration of the medium. **b** Glucose release in dependency of pH of the medium. **c** Glucose release in dependency of temperature. **d** Glucose release in dependency of ammonia concentration in the medium. General experimental conditions: Humidity = 80%, shaking frequency = 970 rpm, shaking diameter = 3 mm, V_L,96_ = 1000 μL/well

The investigation described above revealed that glucose release depends on various parameters. Therefore, a tool to predict the glucose amount released from the matrix into the medium for various media conditions is developed. In Fig. 4, glucose release rates (*v*) for varying osmotic concentrations (A), pH (B), temperatures (C) and ammonia concentrations (D) are presented with symbols. To describe the influence of each parameter, suitable equations are proposed (Fig. 4). The values for the corresponding fitting parameters (a, b and c for each equation) are recorded in Table 1. As depicted in Fig. 4A, the glucose release rates decrease linearly with increasing osmotic concentration (Osmo) in the range between 216 and 582 mOsmol/L. For the pH range from 4.5 to 8, a sigmoidal correlation fits best the measured glucose release rates. In the equation shown in Fig. 3B, *v*_max_ corresponds to the highest glucose release rate measured, which was 0.251 mg/h for pH 4.5.

**Table 1:** Fitting parameters for the equations in Fig. 4

Fitting parameter	Influence of osmotic concentration (Fig. 4A)	Influence of pH (Fig. 4B)	Influence of temperature (Fig. 4C)	Influence of initial ammonia (Fig. 4D)
a	−1.20 · 10^− 4^ (mg·L)/h/mOsmol	1.51 · 10^− 1^ mg/h	1.41 · 10^+ 22^ mg/h	6.30 · 10^−3^ (mg·L)/h/mmol_NH4+_
b	0.164 mg/h	4.22	1.41 · 10^+ 8^	2.07 · 10^− 1^ mg/h
c	–	5.44	7.38 · 10^−2^ mg/h	–

The headlines of each column assign the fitting parameters (a, b and c) to the respective equation of Fig. 4. Inserted into the respective equation, the units of the fitting parameters result in the correct unit for a glucose release rate (mg/h)

The temperature dependency was modeled with an adapted, mechanistic approach proposed by Jost [50], describing the diffusion in solid components. With only three data points, the database for this kind of regression is low. However, due to the practical aim of this work - to predict the glucose release in combination with the mechanistic background of the equation - the correlation is sufficiently accurate (Fig. 4C). The influence of the ammonia concentration on the glucose release rate up to 17.3 mM is described by a linear regression following the equation shown in Fig. 4D. For higher ammonia concentrations, a constant glucose release rate of 0.098 g/L/h is defined. The prediction of the total glucose released after a defined time under various media conditions is given by Eq. 1.

1 $$ {\mathrm{m}}_{\mathrm{released}}=\underset{\mathrm{Step}\kern0.5em 2}{\underbrace{v_{\mathrm{standard}}\cdot \underset{\mathrm{Step}\kern0.5em 1}{\underbrace{\prod \limits \frac{v_{\mathrm{i}}}{v_{\mathrm{standard}}}}}}}\cdot t\underset{\mathrm{Step}\kern0.5em 3}{\underbrace{+{\mathrm{m}}_{\mathrm{i}\mathrm{nit}}}} $$

The glucose release rates for the different impact factors (*v*_i_) are normalized in respect to a release rate under “standard” conditions (200 mM Wilms-MOPS, violet triangles in Fig. 3 and 4; *v*_standard_ = 0.0933 mg/h; m_init_ = 0.901 mg), which is defined as the release into the original Wilms-MOPS medium (Step 1). By multiplying the product of all normalized impact factors with the standard release rate (Step 2) and adding m_init_ (Step 3), the total amount of glucose released at every time-point (m_released_) can be calculated.

**Fig. 5:**
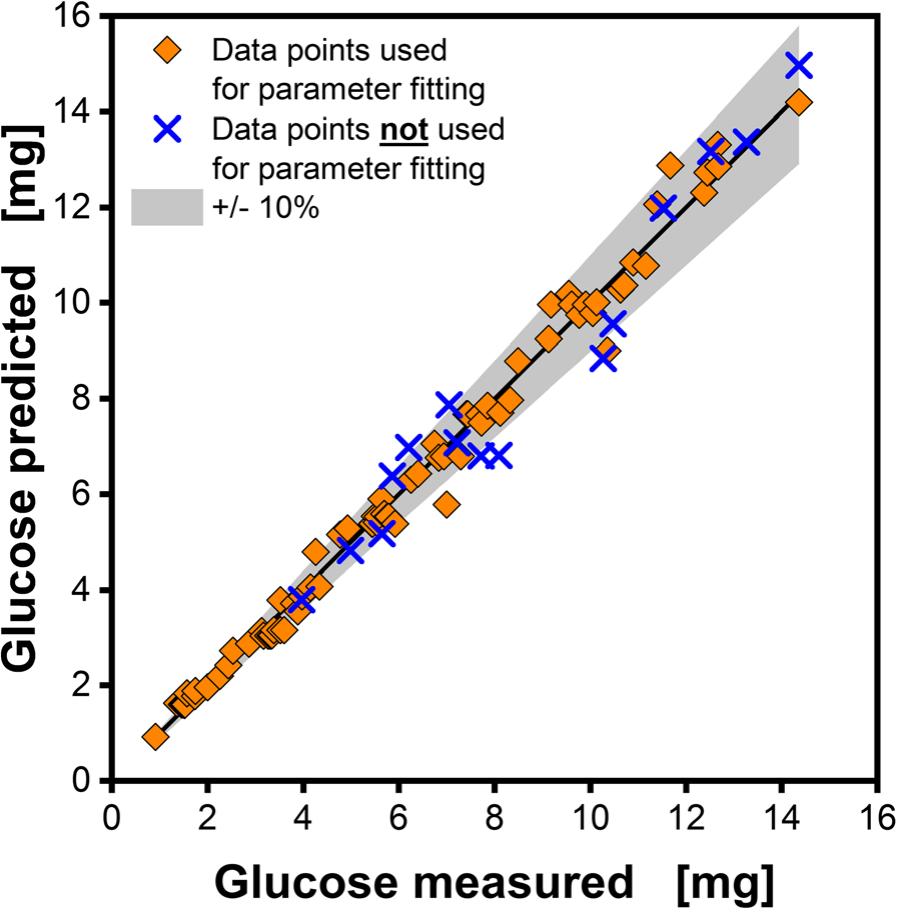
Parity plot of the measured and predicted amount of glucose released per well. Orange diamonds represent the 78 data points in Fig. 3a, b, c and Additional file 1, respectively. These data points are used for parameter fitting. Blue cross symbols represent combinations of osmotic concentration, pH and ammonia concentration, which were not used for parameter fitting following Additional file 2. All data points are mean values of measurements of three individual wells. The black line represents the x = y function and the grey area indicates a 10% deviation range

In Fig. 5 a parity plot of the measured and predicted amount of released glucose is depicted. The orange diamonds represent the 78 data points from Fig. 3A, B, C and Additional file 1, respectively. These data points are used for parameter fitting. The mean standard deviation of all 78 data points was 4.5%. The mean deviation of the prediction from the measured data points was 5.3%. This is illustrated in Fig. 5 by the spacing between the orange diamonds and the solid black line, which represents the y = x function. These results indicate that the glucose release is satisfyingly described by the theoretical assumptions. To verify the predictive power of the tool, parameters were changed to values that were not considered in the tool development. The applied conditions are listed in Additional file 2. Blue crosses represent these experimental results in Fig. 5. Although even multiple parameters were changed at the same time, the mean deviation compared to the measured data was still below 7.3% for in total 16 glucose release data points. This is in the range of the fabrication-related well-to-well accuracy declared by the manufacturer of the plates, which is specified as 10%. Therefore, the prediction error is smaller than the fabrication deviations. Consequently, the developed tool allows for a reliable prediction of the glucose release under various cultivation conditions over time.

The described relations can also explain the higher glucose release of Syn6-MES medium compared to the Wilms-MOPS medium observed in Fig. 2. Syn6-MES medium has a lower pH and a lower osmotic concentration, which both accounts for a higher glucose release. However, YP medium occupies a lower osmotic concentration and pH compared to LB medium, but does not reveal a higher glucose release. Apparently, the predictive tool (Eq. 1) is not directly extendable for complex media.

### Cultivation of a eukaryotic model organism

**Fig. 6:**
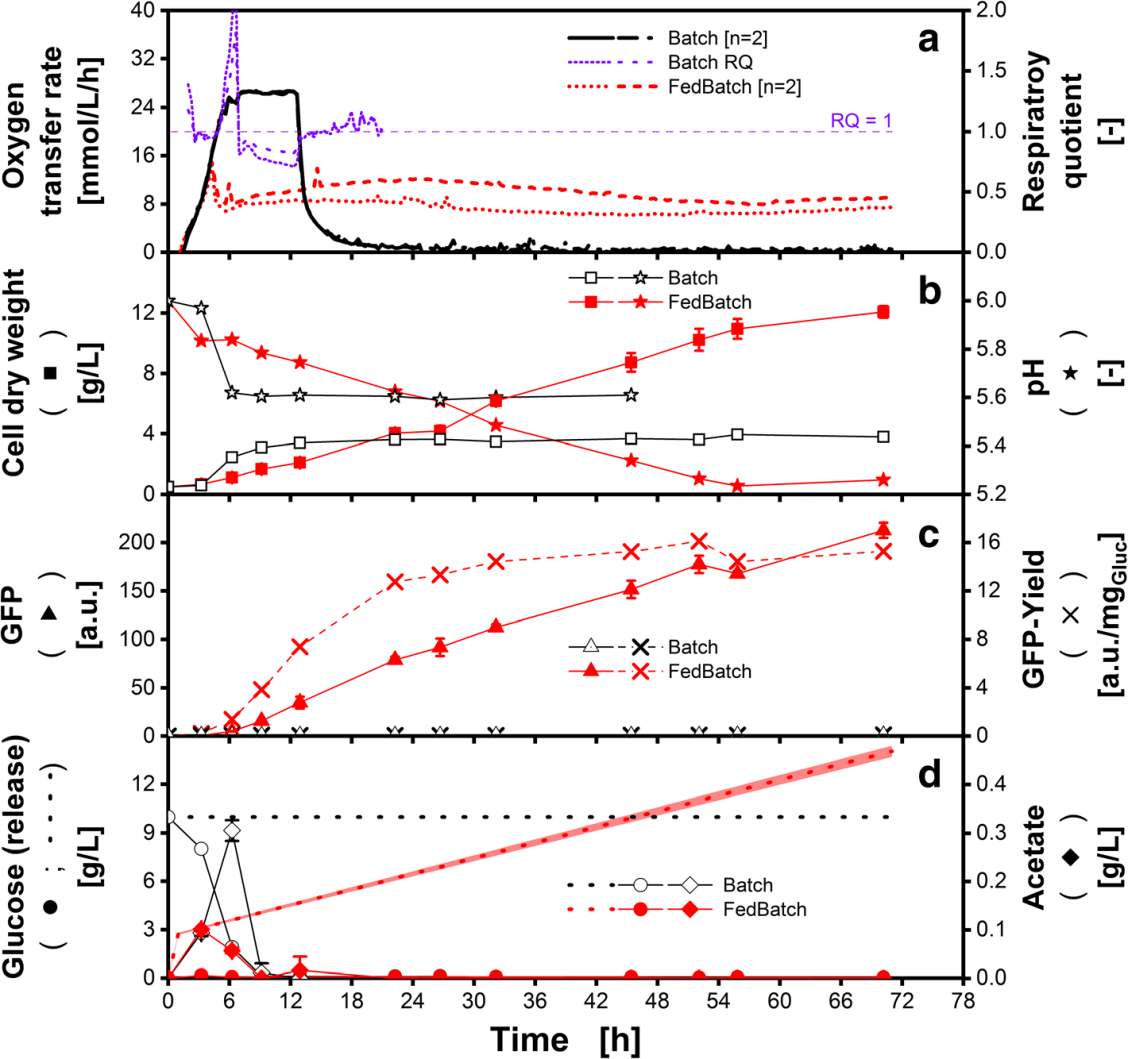
Batch and fed-batch cultivation of *Hansenula polymorpha* RB11 pC10-FMD GFP in 96-square-well microtiter plates. Data correspond to conventional batch microtiter plate (black, open symbols) and in polymer-based fed-batch microtiter plate (red, solid symbols) in Syn6-MES medium. All offline measured data points are mean values of measurements of four individual wells. Exception: Oxygen transfer rate and pH are measured in duplicates. A) Oxygen transfer rate (OTR) and respiratory quotient (RQ) B) Cell dry weight (CDW, squares) and pH (stars); C) Green fluorescence protein (GFP, triangle) and GFP-yield per glucose (cross); D) Measured glucose (circle) and acetate (diamond) concentration. Dotted lines represent the calculated total glucose concentration available for the microorganisms until the respective time. For the calculation (Equation 1), the initial medium properties were applied. Cultivation conditions: initial biomass concentration: 0.35 g/L, temperature = 37 °C; pH_0_ = 6; shaking frequency: *n* = 970 rpm; shaking diameter: d = 3 mm; culture volume V_L,96_ = 600 μL/well; initial glucose concentration c_S_Batch_ = 10 g/L, c_S_FedBatch_ = 0 g/L. Glucose was used as sole carbon source

In Fig. 6 a batch cultivation of *H. polymorpha* RB11 pC10-FMD GFP is illustrated as a benchmark with black symbols and lines. The oxygen transfer rate (OTR) (Fig. 6A) shows an exponential increase within the first 5 h, followed by a short decrease (~ 1 h) and a plateau of another 6 h. Afterwards, the respiratory activity decreases to almost zero. During the first phase of exponential growth, glucose is consumed and biomass is produced (Fig. 6B and C). As soon as the OTR reaches roughly 25 mmol/L/h (Fig. 6A; 6–12 h), the maximum oxygen transfer capacity is reached for these applied conditions, and thus the cultivation is oxygen-limited. In combination with the short OTR decrease, the high respiratory quotient (6–7 h) indicates oxygen limited growth with simultaneous production of a reduced compound, presumable ethanol (not analysed) [35]. Additionally, acetate (Fig. 6D; diamonds) is accumulated to a level of 0.3 g/L as an overflow metabolite whereby a moderate drop in pH is provoked (Fig. 6B). After 7 h of cultivation, glucose is depleted and the metabolization of ethanol and acetate starts, which is indicated by a RQ below 1. The final cell dry weight concentration is approximately 3.6 g/L. For the whole batch process, nearly no GFP is produced. This is caused by an initial repression of GFP production due to high glucose concentrations.

In comparison, the fed-batch cultivation shows a completely different behaviour. The OTR increases exponentially within the first 4 h in parallel to the batch cultivation (Fig. 6A). In this phase, the initial biomass concentration is too small to take up the released glucose straightaway. Thus, glucose accumulates (Fig. 6D) and the OTR profile resembles a short batch cultivation. This characteristic appears also with other simple fed-batch methods like the enzymatic glucose release system, as depicted in Panula-Perälä et al. [30]. After this initial batch phase, glucose becomes limiting and all further released glucose is consumed immediately by the organisms. In Fig. 6A, this results in an OTR plateau of around 7–10 mmol/L/h. Since there was only a short batch phase and oxygen was never limited, only 0.1 g/L acetate were formed (Fig. 6D). The acetate was consumed quickly after entering the fed-batch phase. In the progress of the fed-batch phase, biomass constantly increases until the end of the cultivation. After 22 h, the cell dry weight exceeds the level of the batch cultivation. At this point, the total amount of glucose released (6 g/L) is lower compared to the amount that was provided in the batch cultivation (10 g/L). After 46 h, the glucose released equals the amount of provided glucose in batch culture. In contrast to the sharp pH drop, as it occurs during batch cultivation, a smooth decline in pH is observed during fed-batch cultivation (Fig. 6B). This is due to ammonia consumption accompanied by the secretion of a proton to the media [55]. In fed-batch cultivations, the GFP fluorescence intensity is increased 245 times. Furthermore, the product yield for GFP with respect to glucose is 176 times and - with respect to biomass - 77 times higher compared to the batch cultivation after 79 h (Fig. 6C). In fed-batch mode, GFP expression is derepressed since no elevated concentration of glucose is present. The glucose release in Fig. 6D was calculated by a regression of the data points in Fig. 2B obtained for Syn6-MES medium. In summary, the polymer-based fed-batch microtiter plate was successfully applied for a yeast cultivation with product formation. These results demonstrate that a typical fed-batch cultivation was established in the microtiter plate, showing all characteristics (avoiding overflow metabolites and oxygen limitation, circumventing catabolite repression) of (large-scale) fed-batch cultivation.

### Cultivation of a prokaryotic model organism

**Fig. 7:**
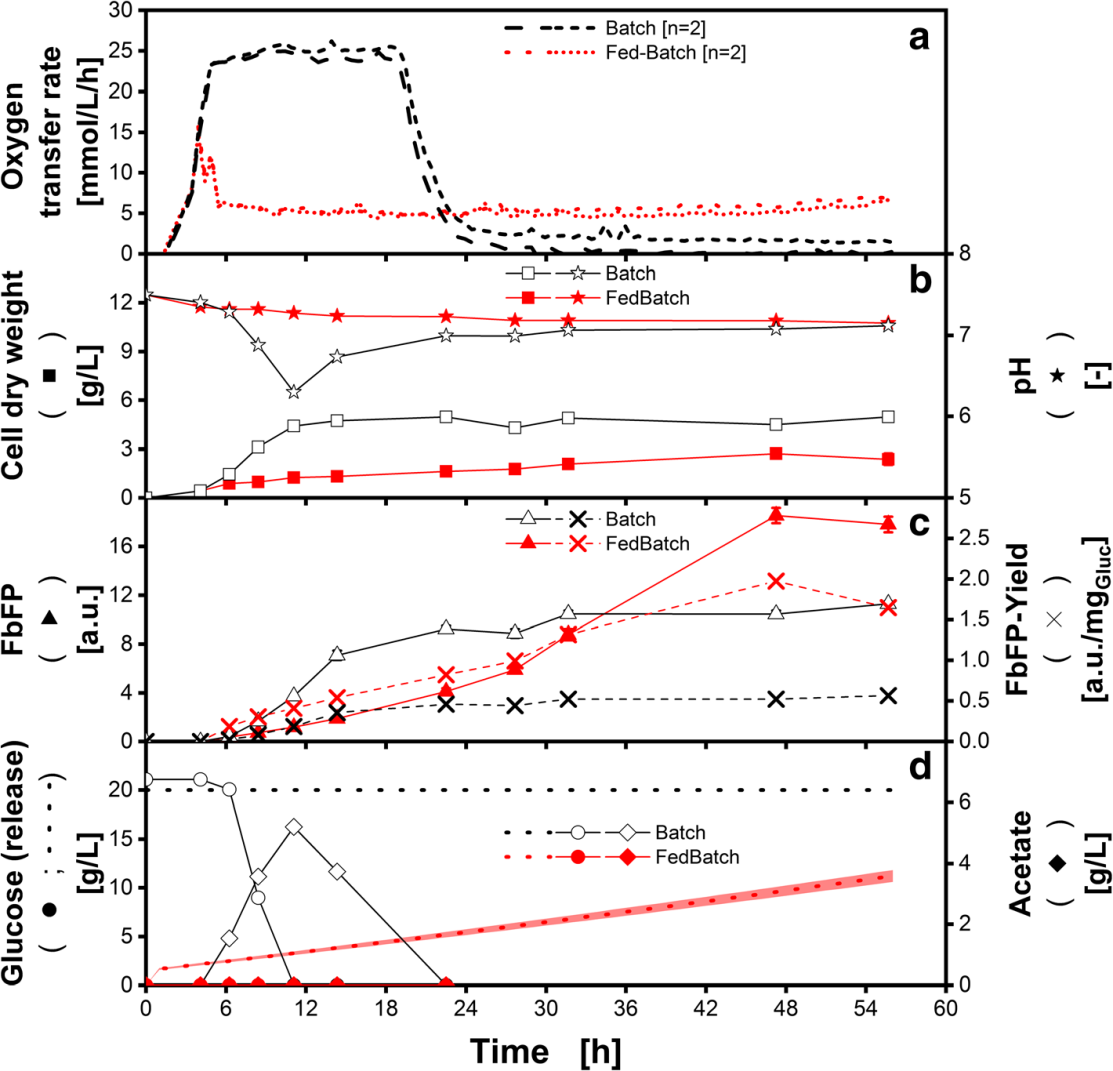
Batch and fed-batch cultivation of *Escherichia coli* BL21 (DE3) pRhotHi-2-EcFbFP in 96-square-well microtiter plates. Data correspond to conventional batch microtiter plate (black, open symbols) and in commercially available polymer-based fed-batch microtiter plate (red, solid symbols) in Wilms-MOPS medium. All offline measured data points are mean values of measurements of four individual wells, the error bars indicate the standard deviation. Exception: Oxygen transfer rate (OTR) and pH are measured in duplicates. **a** Oxygen transfer rate (OTR) **b** Cell dry weight (CDW, squares) and pH (stars); **c** Flavin mononucleotide binding fluorescent protein (FbFP, triangle) and FbFP-Yield per glucose (cross); **d** Measured glucose (circle) and acetate (diamond) concentration. Dotted lines represent the calculated total glucose concentration (Eq. 1) available for the microorganisms until the respective point of cultivation. For the calculation, the initial medium properties were applied. Cultivation conditions: initial biomass concentration: 0.11 g/L, temperature = 37 °C; pH_0_ = 7.5; shaking frequency: n = 970 rpm; shaking diameter: d = 3 mm; culture volume V_L,96_ = 600 μL/well, initial glucose concentration c_S_Batch_ = 20 g/L, c_S_FedBatch_ = 0 g/L. Glucose was used as sole carbon source

In Fig. 7, a batch (black) and a fed-batch (red) cultivation of a *E. coli* BL21 (DE3) pRhotHi-2-EcFbFP in microtiter plates are displayed. In Fig. 7A, the oxygen transfer rate for the batch cultivation appears similar to the one seen in the *H. polymorpha* cultivation (Fig. 6A). In the first 5 h, an exponential increase is followed by a plateau of roughly 14 h. Afterwards, the OTR drops down to zero. Again, the first exponential phase corresponds to oxygen unlimited growth on glucose. Hence, the biomass increases (Fig. 7B), whereas the glucose decreases (Fig. 7D). Upon reaching the OTR plateau of about 25 mmol/L/h after 5 h, glucose is still consumed, but under oxygen limited conditions. This leads to an increased acetate production, and henceforth a lower biomass formation (Fig. 7B and C). Due to the acetate accumulation, the pH drops to a (suboptimal) value of 6.3. After about 11 h of cultivation, glucose is completely consumed and the microorganisms start to assimilate acetate (Fig. 7D), which is also reflected by the increasing pH. The increase in biomass during growth on acetate is small since the biomass yield on acetate is known to be smaller than for glucose [4, 56]. The final biomass concentration is 4.8 g/L, leading to a biomass yield of 0,24 g_Biomass_/g_Glucose_. This relatively low biomass yield is due to the long and intense oxygen limitation forcing the organism to excessive acetate production. The FbFP-yield with respect to the provided glucose is 0.56 a.u./g_Glucose_.

The fed-batch cultivation starts with a short (4 h) initial batch phase, followed by a little acetate peak at 5 h cultivation time. In this phase, no significant increase of FbFP is detected (Fig. 7C). Upon reaching the oxygen unlimited fed-batch phase, indicated through the OTR plateau (5–6 mmol/L/h), a steady increase in biomass and FbFP was observed. In this phase, the microorganisms immediately assimilate all released glucose. The level of the fed-batch OTR plateau is lower compared to the yeast cultivation due to the reduced glucose release provoked through the changed media conditions. Since no elevated glucose concentrations appear in fed-batch, less acetate is produced and the pH does not drop below the optimal range for *E. coli* cultivations (Fig. 7B and D) [48, 49]. The biomass concentration, however, never reaches those values achieved in batch cultivations. This is reasonable, since the total amount of glucose released into the fed-batch culture (red dashed line, Fig. 7D) never exceeds the initial glucose concentration of 20 g/L provided in the batch experiment (black dashed line). In this case, Eq. 1 was applied to calculate the released glucose with the initial media parameter. The FbFP fluorescence under fed-batch conditions outperforms the one of the batch cultivation after about 36 h. The FbFP-yield with respect to glucose is with 1.94 a.u./g_Glucose_ more than three times higher in comparison to the batch experiment.

**Fig. 8:**
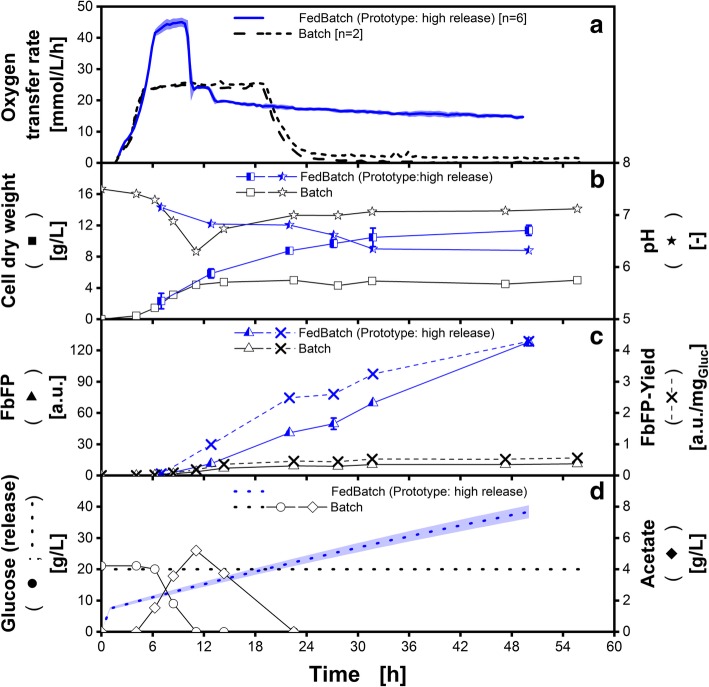
High release fed-batch cultivation of *Escherichia coli* BL21 (DE3) pRhotHi-2-EcFbFP in Wilms-MOPS medium. Data correspond to 96-square-well conventional batch microtiter plate (black, open symbols) and to the newly developed prototype 48-round-well fed-batch microtiter plate for high glucose release (blue, half-filled symbols). All offline measured data points are mean values of measurements of four individual wells, the error bars indicate the standard deviation. Exception: Oxygen transfer rate (OTR) and pH are measured in duplicates. A) Oxygen transfer rate (OTR); the OTR in the newly developed prototype microtiter plate is measured in six individual wells. The blueish shadow indicates the standard deviation of these measurements. B) Cell dry weight (CDW, squares) and pH (stars); C) Flavin mononucleotide binding fluorescent protein (FbFP, triangle) and FbFP-Yield per glucose (cross); D) Measured glucose (circle) and acetate (diamond) concentration. Dotted lines represent the calculated total glucose concentration available for the microorganisms until the respective point of cultivation. Cultivation conditions: initial biomass concentration: 0.11 g/L, temperature = 37 °C; pH_0_ = 7.5; shaking frequency: n = 970 rpm; shaking diameter: d = 3 mm; culture volume V_L,96_ = 600 μL/well, V_L,48_ = 800 μL/well; initial glucose concentration c_S_Batch_ = 20 g/L, c_S_FedBatch_ = 0 g/L. Glucose was used as sole carbon source

To investigate the influence of varying glucose release rates on the productivity, an adapted silicone matrix was applied. In Fig. 8 (blue lines and symbols), a fed-batch cultivation with the 48-round-well prototype microtiter plate system is displayed. Release experiments with this prototype revealed a mean increase in glucose release by the factor of 3.7 with respect to the commercially available fed-batch microtiter plate. This factor already considers the increased polymer surface area. This leads to a glucose release as it is depicted in Fig. 8D. The glucose accumulation in the initial cultivation phase is higher, leading to a prolonged batch phase, visible in the OTR until about 10 h of cultivation time (Fig. 8A). Additionally, due to the distinct glucose accumulation, the cultivation is oxygen limited for 3 h at the end of the batch phase, indicated by the OTR plateau at around 45 mmol/L/h [57]. There is always a trade-off between high glucose release rates and the risk of reaching oxygen-limited conditions. The second, smaller peak in the OTR between 11 and 13 h represents the consumption of produced acetate. The pH drops more pronounced compared to the standard fed-batch plate (Fig. 8B), which is due to an increase in acetate formation and increased uptake of ammonia. Nevertheless, the biomass exceeds the concentrations reached during batch cultivations after roughly 10 h. After about 31 h the biomass increase stagnates. The applied Wilms-MOPS medium was developed for 20 g/L glucose concentrations [58]. Since the prototype fed-batch microtiter plate exceeds this concentration, another nutrient might becomes limiting, thus hindering biomass formation. Alternative, but more complex feeding systems (like e.g. BioLector Pro) achieve even higher glucose feeding rates. However, also in this system biomass saturates with high feeding rates [29]. The final FbFP fluorescence with this system surpasses the performance of the commercial fed-batch microtiter plate by a factor of 7 and the batch cultivation by a factor of 11 (Fig. 8C). The raised glucose release rate provokes a further increased FbFP-yield with respect to glucose to 4.3 a.u./g_Glucose_. Already in this small-scale experiment, it is possible to investigate the influence of different release rates.

In the study of Philip et al. [59] results for a fed-batch cultivation of the same *E. coli* strain in a 1 L bench top stirred tank reactor were presented. The reported data and the results in this work using the polymer-based fed-batch microtiter plate are highly consistent concerning the measured process parameters. This means that the results from the fed-batch microtiter plate are predictable and transferable to bench top stirred tank reactors.

## Conclusion

To meet the requirements of a consistent bioprocess development, the large-scale production conditions need to be imitated in each stage of the development process. Until now, high-throughput small-scale microtiter plate cultivations in fed-batch mode were only feasible with huge effort and considerably high investments or with a sensitive enzymatic release system. In this study, an alternative fed-batch system was thoroughly investigated, enabling a cost effective integration into existing microtiter plate infrastructure, like microtiter plate or liquid handling systems. Therefore, established working procedures do not need to be adjusted and comparability to former results is ensured.

The introduced polymer-based fed-batch microtiter plate enables a linear and reproducible glucose release into the culture medium. The specific glucose release rates are dependent on media conditions and temperature. The prediction of the total amount of released glucose into a defined medium based on a developed tool proved to be highly accurate. The fed-batch cultivation of both, *H. polymorpha* producing GFP and *E. coli* producing FbFP, showed that typical glucose limited conditions, as they occur in industrial processes, were provided. Both organisms cultivated in fed-batch mode outperformed the corresponding batch cultivation with respect to product formation. *H. polymorpha* even produced 245 times more product in fed-batch mode. The potential of the polymer-based fed-batch microtiter plate for early process development is displayed. Other costly feeding systems (e.g. parallel-operated milliliter-scale stirred tank bioreactors) are additionally capable of exponential feeding profiles [23]. Nevertheless, the fed-batch microtiter plate offers the possibility to implement glucose limited fed-batch conditions in a simple, non-expensive manner. Especially, the influence of different glucose release rates on the productivity could be evaluated. In conclusion, the polymer-based fed-batch microtiter plate investigated in this study proved to be a robust and easy to handle tool to generate fed-batch conditions for strain screening and for simplifying and accelerating bioprocess development.

## Additional files

Additional file 1: Total amount of glucose released into Wilms-MOPS medium with varying initial (NH_4_)_2_SO_4_ concentration. (DOCX 120 kb)

Additional file 2: Applied parameters and time points of experiments used for tool prediction. (DOCX 41 kb)

## Abbreviations

e.g.: Exempli gratia, for example
FbFP: Flavin mononucleotide binding fluorescence protein
GFP: Green fluorescence protein
HPLC: High-performance liquid chromatography
LB: Lysogeny broth
OD_600_: Optical density at 600 nm
Osmo: Osmotic concentration
OTR: Oxygen transfer rate
PBS: Phosphate buffered saline
R: Universal gas constant
RQ: Respiratory quotient
TB: Terrific broth
*v*: Glucose release rate
YP: Yeast extract peptone
