# Supplementary material for: Polymer-based controlled-release fed-batch microtiter plate – diminishing the gap between early process development and production conditions
Source: J Biol Eng. 2019 Feb 22;13:18. doi: 10.1186/s13036-019-0147-6 (PMC6387502; doi:10.1186/s13036-019-0147-6)
Supplement: Supplementary file 1 — Total amount of glucose released into Wilms-MOPS medium with varying initial (NH4)2SO4 concentration. (DOCX 120 kb) [file 13036_2019_147_MOESM1_ESM.docx]

**Additional file 1**





Additional file 1

**Total amount of glucose released into Wilms-MOPS medium with varying initial (NH_4_)_2_SO_4_ concentration.** Glucose release per well of a 96-square-well fed-batch microtiter plate is depicted after 24 h (black) and 48 h (purple). All data points are mean values of measurements of three individual wells. Error‑bars indicate the respective standard deviation. The indicated glucose value of 0.901 mg represents the mean y-intercept of all linear fittings of the data presented in Figure 3. Experimental conditions: 200 mM MOPS buffer, osmotic concentration = 582 mOsmol/L, temperature = 37 °C, pH = 7.5, humidity = 80 %, shaking frequency = 970 rpm, shaking diameter = 3 mm, V_L,96_ = 1000 µL/well. No biology was applied. To avoid unintended growth of contaminants, 0.2 g/L NaN_3_ was added to the medium.
