# Supplementary material for: Polymer-based controlled-release fed-batch microtiter plate – diminishing the gap between early process development and production conditions
Source: J Biol Eng. 2019 Feb 22;13:18. doi: 10.1186/s13036-019-0147-6 (PMC6387502; doi:10.1186/s13036-019-0147-6)
Supplement: Supplementary file 2 — Applied parameters and time points of experiments used for tool prediction. (DOCX 41 kb) [file 13036_2019_147_MOESM2_ESM.docx]

**Additional file 2**

Applied parameters and time points of experiments used for tool prediction.

| **Time [h]** | **Osmotic concentration [mOsmol/L]** | **Initial pH [-]** | **Initial ammonia [mmol/L]** |
| --- | --- | --- | --- |
| 24 | 216 | 6 | 105 |
| 24 | 311 | 6 | 105 |
| 24 | 347 | 7 | 105 |
| 24 | 316 | 5.5 | 105 |
| 24 | 390 | 6 | 3.28 |
| 24 | 392 | 6 | 6.56 |
| 24 | 418 | 5.5 | 105 |
| 24 | 383 | 6.5 | 105 |
| 48 | 216 | 6 | 105 |
| 48 | 311 | 6 | 105 |
| 48 | 347 | 7 | 105 |
| 48 | 316 | 5.5 | 105 |
| 48 | 390 | 6 | 3.28 |
| 48 | 392 | 6 | 6.56 |
| 48 | 418 | 5.5 | 105 |
| 48 | 383 | 6.5 | 105 |
